# Veratrum Virida in Pneumonia

**Published:** 1870-10

**Authors:** 


Veratrum Virida in Pneumonia.—Dr. T. M. Woodson, in the *American Practitioner*, strongly advocates the use of veratrum in pneumonia. He gives a history of four cases in which he used it in six to seven drop doses every three or four hours, with large doses of Dover powder, and sometimes calomel. Some of these cases were very severe, the pulse reaching 132, with the temperature 105°, and respiration over 30. The drug brought down the pulse promptly, sometimes with vomiting and purging, but safely, to from 55 to 70 per minute; the temperature and respiration were proportionally reduced, and convalescence was speedily established. The doctor has followed this practice for many years, with fine success. The veratrum works more kindly in conjunction with opium, and these two agents he places at the head of all remedies for the active stage of pneumonia.

